# Nuclei deformation reveals pressure distributions in 3D cell clusters

**DOI:** 10.1371/journal.pone.0221753

**Published:** 2019-09-12

**Authors:** Adele Khavari, Allen Joseph Ehrlicher

**Affiliations:** 1 Applied Chemistry, Chemistry and Chemical Engineering, Chalmers University of Technology, Göteborg, Sweden; 2 Department of Bioengineering, McGill University, Montreal, Canada; Universitat Zurich, SWITZERLAND

## Abstract

Measuring pressures within complex multi-cellular environments is critical for studying mechanobiology as these forces trigger diverse biological responses, however, these studies are difficult as a deeply embedded yet well-calibrated probe is required. In this manuscript, we use endogenous cell nuclei as pressure sensors by introducing a fluorescent protein localized to the nucleus and confocal microscopy to measure the individual nuclear volumes in 3D multi-cellular aggregates. We calibrate this measurement of nuclear volume to pressure by quantifying the nuclear volume change as a function of osmotic pressure in isolated 2D culture. Using this technique, we find that in multicellular structures, the nuclear compressive mechanical stresses are on the order of MPa, increase with cell number in the cluster, and that the distribution of stresses is homogenous in spherical cell clusters, but highly asymmetric in oblong clusters. This approach may facilitate quantitative mechanical measurements in complex and extended biological structures both *in vitro* and *in vivo*.

## Introduction

Cells are exposed to diverse forces *in vivo*, including compression, tension, fluid shear. These stimuli are transduced into biochemical signals, which in turn regulate cell mechanics and behavior. New mechanotransduction interactions are being discovered, but some known mechanisms include elements that connect the cell to its environment through focal adhesions[1, 2], the actin cytoskeleton which measures crosslinker strain [3]. Measuring the cellular forces and mechanics that drive mechanotransduction *in vitro* and *in vivo* is an integral part of modern quantitative biology, and a broad variety of techniques to measure cell forces and mechanics have been developed, particularly in the last two decades. Traction Force Microscopy has emerged as an excellent measure of cellular contractile forces in 2D [4, 5] and 3D [5], while AFM has been used to measure aspects such as the protrusive forces [6] and local cortical mechanics of cells [7]. Additionally, magnetic twisting cytometry has proven to be a rapid method to measure the cortical mechanics of many cells simultaneously [8]. These techniques all share in common the approach of requiring direct mechanical contact with the cell to perform the measurement; techniques to measure distal components, such as the interior cytoplasmic moduli [9] and forces [10] of cells preclude the use of methods such as AFM, and highlight the need for non-contact techniques such as optical tweezers [11]. Moreover, these approaches have been mainly used for isolated cells in *in vitro* systems, and are not readily applicable to more complex 3D structures of multiple cells and complex mechanical structures. Recently an approach has combined microrheology and optical tweezers to infer extracellular matrix stiffening from cell contractility[12]. Other groups have used the deformation of fluid drops to measure the local anisotropic stresses *in vivo* in tissues [13]. This fluid-drop approach provides detailed information about the stresses between cells, but it does not readily address isotropic or compressive forces, and requires the introduction of an external force probe.

Compressive stresses are of particular interest as they can lead to nuclear deformation[14] As the largest organelle and genetic control center of the cell, the nucleus is particularly sensitive to external forces [15], which can change influence gene expression[16–18]. Therefore, quantifying local stresses on individual nuclei is a critical aspect of understanding mechanotransduction. Recently researchers have introduced polyacrylamide particles in attempts to measure compressive forces[19] and alginate spheres [20], and while these methods offer insight into local compressive forces, they face challenges including requiring external probes.

An ideal probe for compressive stresses would be an endogenous, ubiquitous, uniform, and dispersed collection of compliant spheres. Indeed, the nucleus itself presents a large spheroid, with a generally constant size in normal mammalian cells, have very similar sizes within a given population for many cells [21–23], and while cell nuclei are not perfectly spherical they are typically singular, and have a constant volume throughout the majority of the cell cycle[21]. These aspects make them appealing as potential targets for use as endogenous sensors. Supporting this idea, in 2D environments, nuclei have been shown to have a very predictable response to applied pressures.[16, 17, 24–27]; here, we build on this previous work and extend the use of nuclei as endogenous pressure sensors to three-dimensions and complex multi-cellular environments.

## Material and methods

### Cell culture

NIH3T3 *Mus musculus*, mouse (Passage 5–30) cells (ATCC) and M6 cells (C. Jorcyk, Boise State University) were grown in culture medium with 10% FBS and 1% antibiotic (pen strep). M6 cells were transfected with Nuclear Location Sequence tagged with Green Fluorescent Protein (BD Biosciences, modified pEGF-C1 with NLS-GFP) using Lipofectamine 2000 (Invitrogen) following Invitrogen’s protocol. After transfection, successfully transfected cells were isolated by FACS (MoFlo XDP Cell Sorter), and growth of untransfected cells was suppressed by adding a selective antibiotic (G418, Invitrogen) to the growth medium. After three days of selection, the positively transfected cells were grown in full medium. Before volume measurements, cells were trypsinized and resuspended in the 3D sodium alginate gels, creating an environment with readily tuned physiological moduli, capable of supporting cell growth and proliferation for several weeks. As these cells proliferate, they form spheroid multi-cellular aggregates (MCAs), a very simplified analog for complex tissue structures (detailed MCAs growth described in [28]).

For single cell volume measurements cells were trypsinized, washed, centrifuged in full culture medium, and resuspended in full medium with different concentration of polyethylene glycol (PEG1500, Sigma), cells were allowed to equilibrate for 20 minutes at room temperature, then plated on a very thin layer of agar gel on the glass bottom petri dishes to avoid adhesion. Nuclei in single-cell pressure-volume calibration measurements were stained with DAPI (Bisbenzimide—Sigma) and reconstructed from confocal images (Leica SP8) using imageJ (imagej.com).

### Microscopy, image processing, and image analysis

Single cell nuclei (stained with DAPI (Bisbenzimide—Sigma)) images, were acquired using a 40x air with NA = 0.75 objective on an inverted confocal microscope (LSM Zeiss 700 or Leica TCS SP8). Each individual cell nuclei was imaged precisely by acquiring XYZ stacks with a 0.5 μm Z step and XY resolution of (72.73x72.73μm). Cells were plated on a thin layer of agar gel to avoid adhesion during imaging.

Images of nuclei within MCAs were acquired using a 10x objective on an inverted confocal microscope (Leica TCS SP5) with a 5μm Z step size. Using this larger step size did not decrease our volume resolution (demonstrated in S2A Fig). To visualize cell nuclei, we transfected cells with Nuclear Location Sequence conjugated with Green Fluorescent Protein (BD Biosciences, modified pEGF-C1 with NLS-GFP).

Image analysis was carried out using ImageJ and the nuclei volumes were calculated by counting voxel number after thresholding the stacks. Nuclei which were not clearly separable in imaging were not considered for analysis.

As objectives are designed to operate in a media of a specific refractive index, the measured z distance is only accurate if the sample matches that expected index. For non-immersion objectives, a refractive index of 1 is expected, whereas cells have a refractive index of approximately 1.37 [29]. We thus correct the measured z values by multiplying the measured z distance by the ratio of true to expected refractive index, i.e. 1.37/1/. This correction was applied for all volume measurements as appropriate for each objective. While we believe that this correction may be neglected when considering relative volume changes using the same objective and microscope, it is essential for determining the relative deformation in X,Y, and Z, absolute volume changes, and when moving between microscopy systems; neglecting it may result in erroneous measurements.

The strains along minor and major axes for nuclei within the MCAs were calculated by measuring the geometry and volume of those nuclei, and comparing them with the undeformed nuclei in isolated single cells in the same alginate gel.

### Calibration of nuclear volume -pressure

#### Osmotic pressure

Hyperosmotic pressure was applied to cells in suspension by adding PEG1500 (Sigma) to isotonic culture medium. The osmolality of different PEG solutions was measured using a freezing point depression osmometer (Advanced Instruments, 3320 Micro-Osmometer), and we then calculated the resulting osmotic pressure (S1 Table). These empirical values are in good agreement with those previously published [30, 31].

We have employed osmotic pressure due to steric exclusion to create our calibration curve for pressure-volume relationship [25]. It is known classically that the nucleus is a porous, permeable membrane-enclosed organelle which allows free movement of water, ions and small molecules through nuclear pore complexes that control the large molecules transfer and act as a molecular sieve. When a porous gel is exposed to an external solution of macromolecules higher than that in the gel, the difference in concentration is quantified by the partition coefficient[32], which leads to an osmotic gradient that draws water out of the gel. This leads to a higher pressure on the gel when the concentration of the macromolecule increases in the solution. Osmotically-induced volume changes of the cell lead to higher intracellular macromolecule concentration which compress the nucleus. Therefore, when we apply hyperosmotic solutions to the cell, these forces are transferred to the nucleus. It is also shown [33] that nuclear compression under hyper osmotic conditions is proportional to intracellular macromolecule concentration, increased molecular crowding within the nucleus. This is similar for both isolated nuclear and the ones in the cell in same osmotic condition, validating our pressure-volume calibration curve.

**Fig 1:**
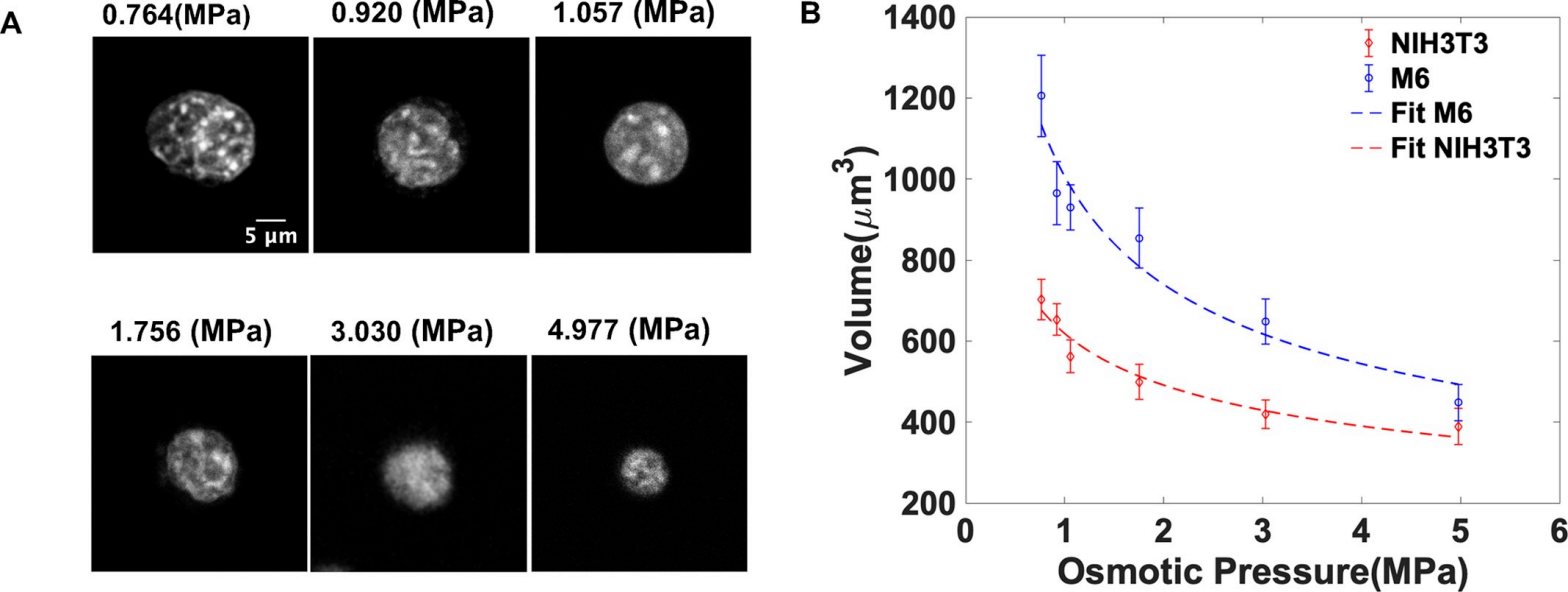
Cell nuclear volume decreases under increasing osmotic pressure. A) 2D cross sections of confocal microscopy images of EGFP-NLS in M6 nuclei in different PEG solutions corresponding to osmotic pressures of 0.764, 0.920, 1.057, 1.756, 3.033, 4.977 MPa; scale bar is 5μm. B) the dependence of nuclear volume on osmotic pressure as measured by reconstructing confocal stacks of EGFP-NLS fluorescent nuclear for different cell types with corrected z values. Error bars are standard deviation. These measurements were done for between 20 and 40 cells for each data point.

We have plotted the measured volume as a function of osmotic pressure applied using PEG (Fig 1B). A single power law regression curve is used to describe the pressure-volume (PV) relationship[31]. This PV curve was subsequently used as a calibration of how nuclei are compressed as a function of pressure allowing us to calculate the local pressures in the MCAs based on the measured nuclear volumes. The governing regression equation and the corresponding coefficients (with 95% confidence bounds) for M6 and NIH3T3 cells are given below,

$$V(P)=a∙P^{b}$$

where for M6 cell a = 1006 ±100 and b = -0.4441±-0.18; for NIH3T3 cell a = 618.6 ± 60 and b = -0.3328±-0.11.

## Results

### Nuclei reversibly and predictably compress under hyperosmotic pressure

Nuclear volumes were measured by direct microscope observation after exposing M6 (n = 35) or NIH3T3 (n = 40) cells to a wide range osmotic pressures via the addition of 1500-Da PEG, as described in the Methods section. As reported previously [16, 23], the nuclear volume decreases with increasing external osmotic pressure, as shown in Fig 1A.

**Fig 2:**
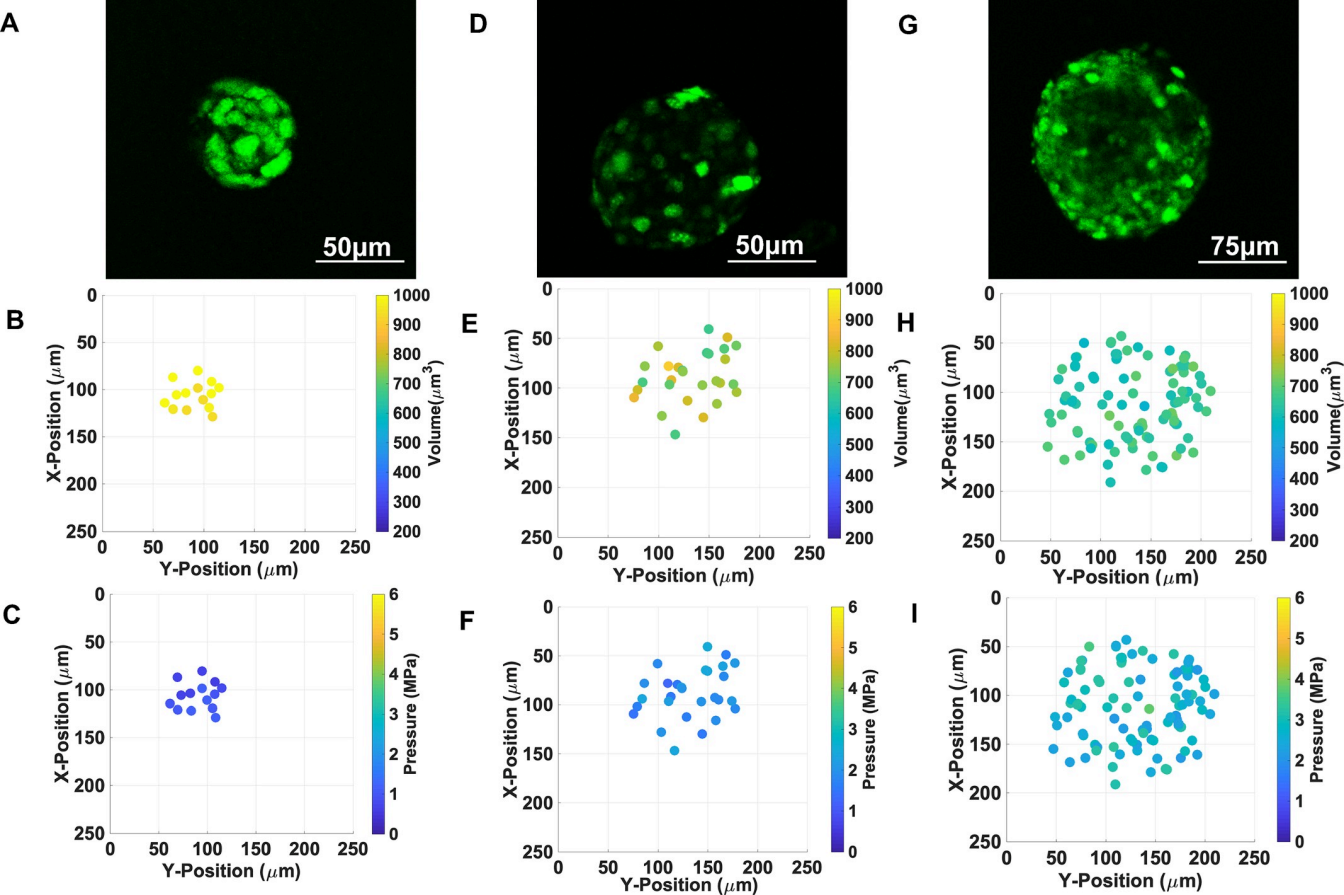
Individual cell nuclei are increasingly compressed as MCAs grow in size. Confocal microscopy images of EGFP-NLS fluorescence of individual nuclei within spherical MCA MCAs with volumes of A) 140+/-10x10^3^, D) 800+/-70 x 10^3^, and G) 2100+/-100 x 10^3^ μm^3^. Nuclei volumes in MCAs with volume of B) 140+/-10x10^3^, E) 800+/-70 x 10^3^, and H) 2100+/-100 x 10^3^ μm^3^. C) corresponding pressure in MCAs with volume of C) 140+/-10x10^3^, F) 800+/-70 x 10^3^, and I) 2100+/-100 x 10^3^ μm^3^ relative to their position in the MCA.

The fitted curve (blue) in Fig 1B of the PV relationship for M6 cells *V*(*P*) = 1006∙*P*^−0.4441^ allows us to calculate the corresponding pressure for the measured nuclei volume in multicellular aggregates (MCA) [33], and depict the local pressure acting on nuclei distributed throughout the MCAs, as shown in Fig 2. Previous work has shown that osmotic stress deforms the cell and nucleus in the same fashion as cytoskeletal contractility[31].

We also find no lasting effects on cells after prolonged exposure to investigated osmotic pressures; after 38 hours of exposure to varying concentrations of PEG, all nuclei recovered to previous control volumes (see S1B Fig), as reported previously[16]. Moreover, the compressibility of nuclei does not change after prolonged exposure to compression; after culturing cells for 21 days under 0.92 MPa, and immediately measuring their PV curve, we found no difference from the PV curve of cells cultured under isotonic conditions.

### Nuclei are more compressed as MCAs grow in size

**Fig 3:**
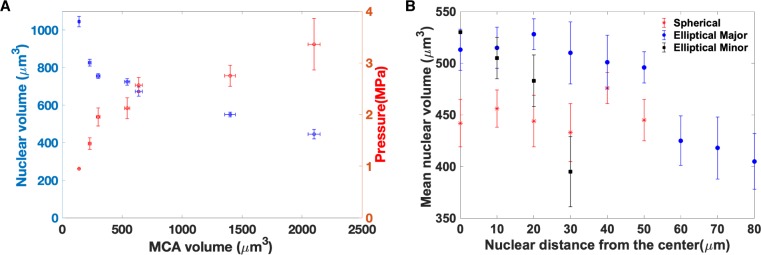
**A) Nuclei are more compressed as MCAs grow in size.** Nuclear volume as a function of MCA volume in spherical MCAs. The number of nuclei range from 20 to 90 in each MCA; Plotted values are mean volumes, with error bars depicting standard deviation. Each data point represents the total data from 2 to 4 MCAs. As the MCAs grow in size, the average nuclear size decreases with a commensurate increase in average pressure. **B) Nuclei in elliptical MCAs are less compressed along the MCA major axis, as compared to the minor axis, or that in spherical MCAs.** changes in elliptical multicellular aggregate from the center towards the edges of the MCAs. This figure presents the volume of the nuclei as a function of position in the MCA; volume of the nuclei in the: spherical (red stars) MCAs, elliptical MCAs from the center along the major axis (blue circles), elliptical MCAs from the center along the minor axis (black squares). Data are taken from one elliptical and one spherical (both 2100+/-100 x 10^3^ μm^3^ volume) MCA, and represent mean and standard deviation.

Nuclei transfected M6 cells were encapsulated in alginate gels and imaged over the course of three weeks, with cell colonies forming multicellular aggregates (MCAs). The volume of nuclei within MCAs of different sizes were imaged with XYZ stacks employing a 5 μm Z step. Number and volume of nuclei depends on the MCA size, where smaller MCAs are composed of cells with larger nuclei. As MCAs grown is size, the average volume of individual nuclei decreases (Fig 3A). Three MCAs with volumes of 140+/-10 x10^3^, 800+/-70 x 10^3^, and 2100+/-100 x 10^3^ μm^3^ are shown in Fig 2A and 2D, and G respectively, and the corresponding nuclei volume and number of nuclei are presented in Fig 2B, 2E and 2H; the volume of the nuclei in biggest MCA is around half of the nuclei volume in smallest MCA. The corresponding pressure of the nuclei in each MCA also shows that nuclei in larger MCAs are bearing higher pressures (Figs 2C and 3A).

### The pressure distribution appears spatially uniform in spherical MCAs

**Fig 4:**
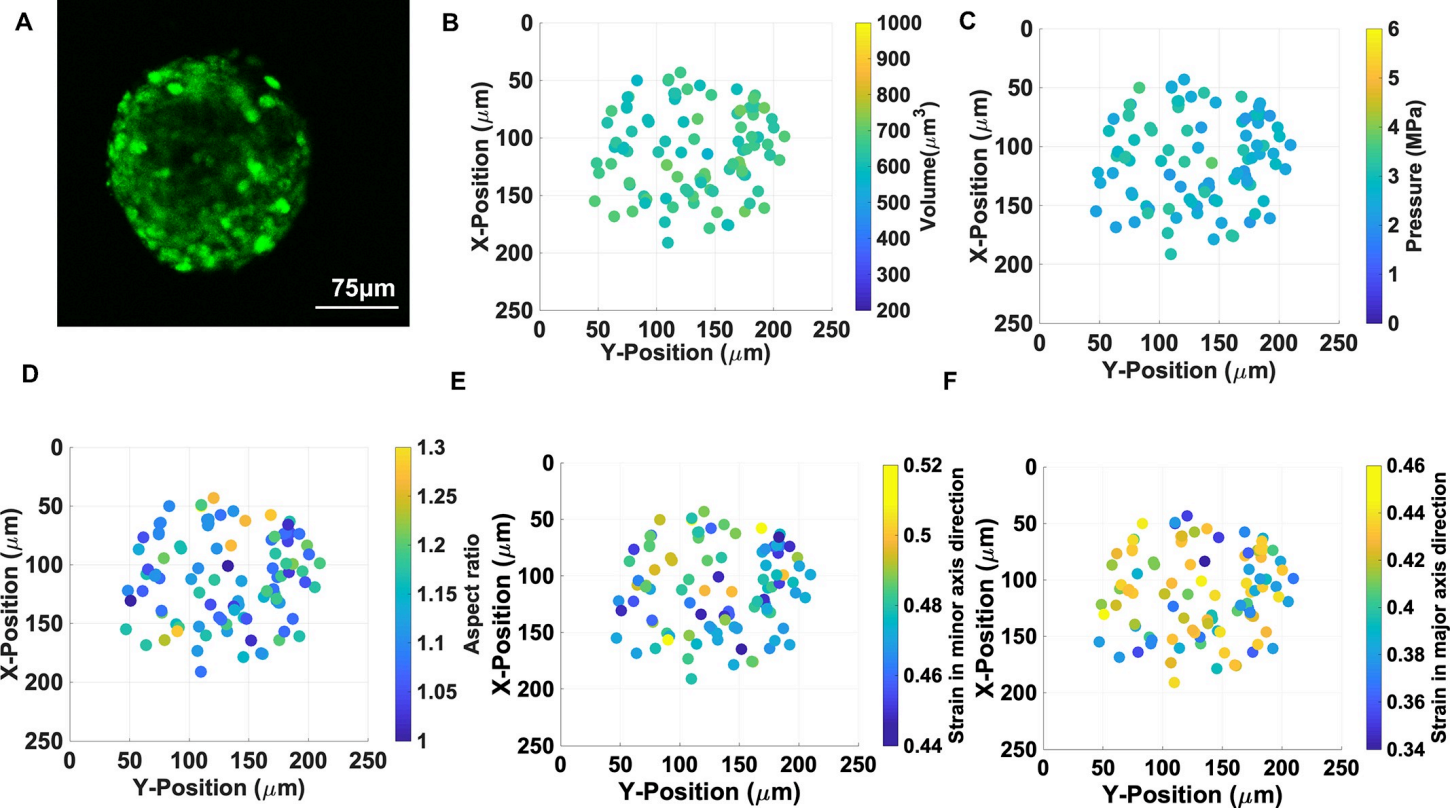
Cell nuclei in spherical multicellular aggregates display stress with no clear relation to their spatial position. A) EGFP-NLS fluorescence image of individual nuclei within a spherical MCA, B) Confocal microscopy determined nuclear volumes, C) Calculated pressures of individual nuclei in the MCA, D) aspect ratios of individual compressed nuclei, E) strain in the direction of minor axis of each nucleus, F) nuclear strains in the direction of each nucleus’s major axis. Comparing strains in panels E & F reveals the anisotropy of compression in spherical MCAs.

As the MCAs grow, they compress their microenvironment; these forces also compress the MCAs, the individual cells, and their organelles as well. We precisely imaged a spherical MCA with the volume of 2100+/-100 x 10^3^ μm^3^ (Fig 4A) in different z-plane and measured the volume of the nuclei within the MCA, which is shown in Fig 4B. The nuclei volumes vary between 595–700 μm^3^ which shows quite a narrow size distribution, and they are uniformly spatially distributed in the MCA. The corresponding pressure for each nucleus calculated based on volume-pressure relationship in Fig 1B shows around 1 MPa difference all over the MCA, however, it is not strongly position dependent, as shown in Fig 4C. We also measured the deformation of nuclei in the direction major and minor axes, which shows nuclei are more compressed in the major axes shown in Fig 4E and 4F. This suggests that the pressure is rather evenly distributed in the investigated MCAs when they have spherical morphologies.

### Nuclei experience higher pressure along the minor axis of elliptical MCAs

**Fig 5:**
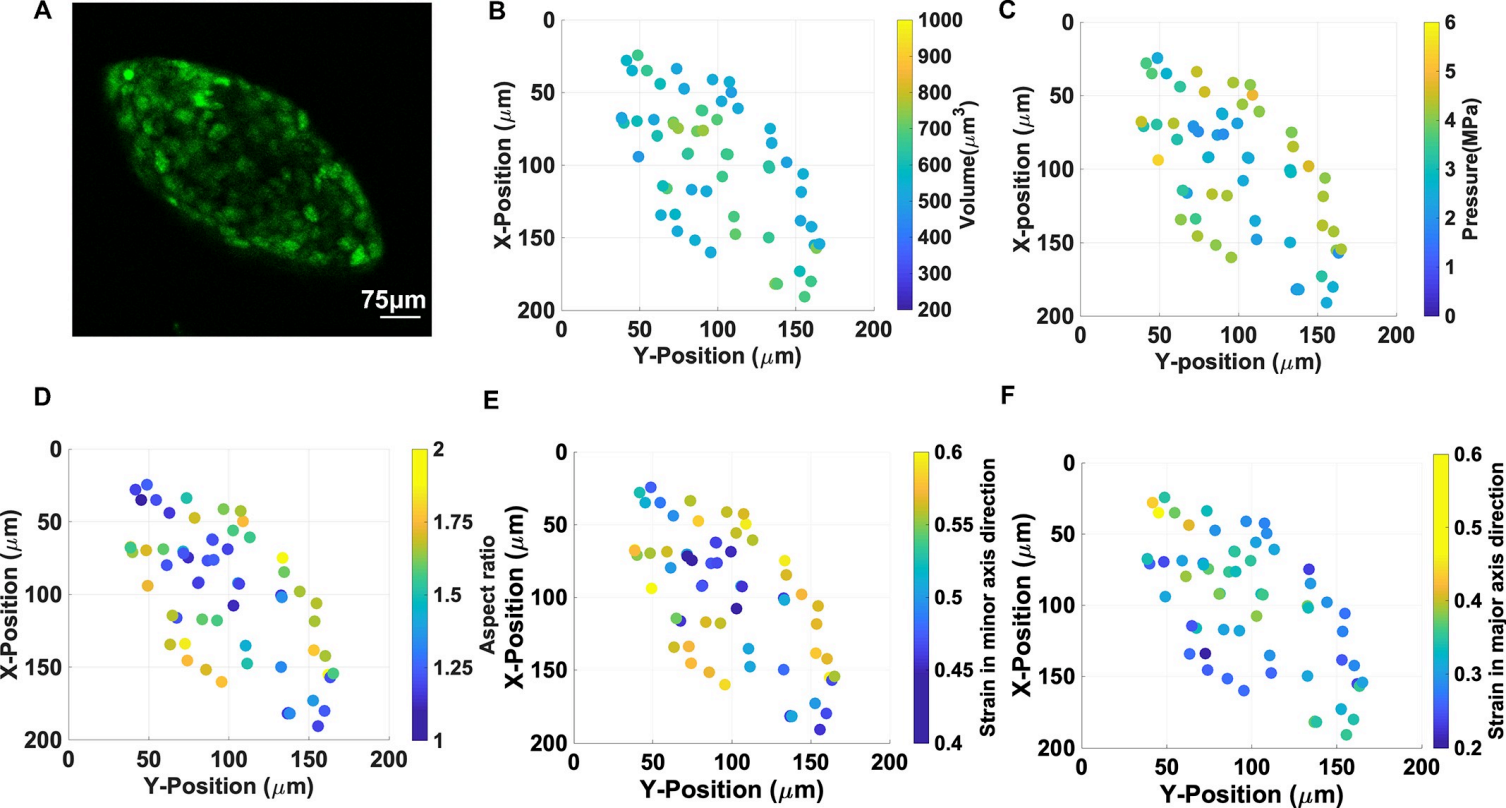
Cell nuclei are compressed more in elliptical multicellular aggregate in the direction of the minor axis of the MCA. A) EGFP-NLS fluorescence image of individual nuclei within an elliptical MCA. B) Confocal microscopy determined nuclear volumes throughout the MCA, C) Calculated pressures of individual nuclei in the MCA, D) aspect ratios of individual compressed nuclei, E) nuclear strains in the direction of minor axis of each nucleus, F) nuclear strains in the direction of major axis of the nuclei. Comparing strains in panels E & F reveals the anisotropy of compression in elliptical MCAs.

In stiffer alginate environments, MCAs tends to grow elliptically (asymmetrically)[28]. We observed that the nuclei remarkably differ in size and shape in elliptical MCA, as shown in Fig 5B and 5D, as a function of position in the MCA, and their shape tends to reflect the overall MCA geometry. Nuclei oriented along the minor axis sides are more elongated and have smaller volumes while those in the center are larger Fig 3B and morphologically closer to spheres. Fig 5C shows that the pressure is also higher at the minor axis and decreases towards the center of the MCA. These measurements are consistent with previous findings which also observed that the local external stress field is higher at the minor axis side of the elliptical MCAs using embedded tracer particles in the gel [34].

## Discussion and conclusion

Here we have shown that nuclei volumes imaged with confocal microscopy may be used as pressure sensors within multicellular aggregates, enabling previously inaccessible regions of multicellular structures or tissues to be measured without the introduction of an exogenous mechanical probe.

We have shown that using PEG we can apply a precise osmotic pressure to produce a reversible and predictable compression of the nucleus. Osmotic pressures from PEG were verified through direct measurement and found to be consistent with literature values. We employed osmotic pressure to measure the pressure-volume (PV) curve of nuclei in isolated cells, where cells are not under compression from neighbors or from substrate-based contraction. The measurement of bulk moduli for cells from osmotic compression is an established technique. It is important to note that unlike physiological salts, there are no pumps for PEG, it does not pass through the membrane, and cells are unable to adapt osmotically; thus, using osmotic stress from a salt-based source would be inaccurate, however, PEG-based osmotic stress is accurate in calculating bulk moduli.

We evaluated the validity of this approach by applying prolonged various pressures to cells, and quantifying their mechanical responses. This demonstrates that these nuclei do not mechanically adapt to these pressures, validating their use as calibrated pressure probes. While these data demonstrate that this approach is robust for measuring these cells and employing them as pressure sensors, this may not always be the case; during early development, differentiation, and disease progression nuclear mechanics may be variable as cells change expression of proteins such as lamins [35–38]. Other conditions such as biochemical, nutrient, or oxygen deprivation may influence bulk moduli. Such mechanical variability of nuclei could hinder or invalidate this methodology.

Having established and validated these PV curves, we then calculate the equivalent total pressure in the multi-cellular aggregate from the confocal microscopy measured volumes.

Our study reveals that these compressive stresses acting on nuclei are in the MPa range, which appears quite large when considering the typical stress regimes of kPa that are reported for cell stresses. A key difference here is the application of compression rather than shear, as bulk moduli [31](~MPa) are 3–4 orders of magnitude larger than shear moduli (~kPa) both for cells, and for inert microgels [39]. This is due to much larger forces being required to compress hydrogels and expel water, than to shear and deform them with no change in volume. As bulk moduli are so large, even small changes in volume represent large compressive stresses on the order of MPa.

The pressures measured here are the sums of all forces compressing the nuclei; these include compressive-stresses, as well as any osmotic pressures from diffusive gradients. We are unable to differentiate between these; as such, we report these as cumulative pressures contributing to nuclear compression. Nevertheless, previous work has shown that compression stresses applied to cells such as through contractility and osmotic pressures applied through diffusive factors such as PEG provoke identical physical and biochemical responses from cells, suggesting that total pressure is a metric mechanosensed by the cell and that compression from any source was sufficient to regulate stem cell differentiation[31]. This highlights the importance of this type of data. To our knowledge, these data represent the first measurements of intracellular compressive forces. Given the importance of nuclear compression in determining cell function and fate, we believe it is of value to the community.

Using these nuclear sensors, we find that stresses compressing nuclei are extremely cell cluster size dependent. Nuclei are more compressed in larger MCAs, suggesting that cell density and MCA volume affect nuclei deformation and compression. As cells proliferate and MCAs grow, the cells compress each other and their microenvironment; higher numbers of cells apply larger stress in the larger MCAs, therefore cells are more deformed and compressed. Our observation is consistent with the work of Nia et al. where they observed an increased tumor size leads to elevated compressive stress [40].

The nuclear stresses are also dependent on MCA geometry, with spherical cell clusters displaying very uniform stresses of approximately 3 MPa throughout the cluster. In stark contrast, elliptical MCAs have highly non-uniform nuclear stress-distributions, with nuclei along the outer major axis bearing roughly twice the pressure (5–6 MPa) of cells in the interior, or at the distal tips of the MCA. Previous work has shown that the MCAs tend to grow in the regions of lower stress[28, 41], suggesting that the lower pressure at the curvature than the sides may be a reason for MCAs to grow elliptically. The nuclei along the sides of the MCAs are deformed in the minor axes much more than the major axis, again mirroring the overall MCA shape.

Differential pressures such as these may play a key role in mechanically regulating cell growth and proliferation in a variety of contexts from development to pathology, as nuclear deformation leads to changes in genes expression and cell function [42], making the nucleus a central mechanosensor for the cell [43]. We hope that this simple technique will enable future work to examine not only how growth changes these pressures, but how these pressures regulate multi-cellular structure and mechanotransduction.

## Supporting information

S1 Table Applied Osmotic pressure on suspended cells plated on thin agar gel (1). Different PEG concentration w/v% in culture media (column 1), create varying osmotic pressure (column2). The osmotic pressure in column 2 corresponds to each PEG concentration based on the literature [1]. The osmolality of different PEG concentration (column 3) was measured by a freezing point depression osmometer, and their corresponding calculated osmotic pressures (column 4) is consistent with values previously reported in the literature. Columns 5&7 are the nuclear volumes for M6 and NIH3T3 cells under different PEG concentration, and columns 6 & 8 are their respective corresponding bulk moduli.
(TIFF)

S1 Fig Osmotic compression does not permanently impact nuclear size. A) Volume of the nuclei measured after culturing cells under an osmotic pressure of 0.920 MPa for 21 days, without osmotic recovery, immediately exposed them to higher pressures. Nuclei that have been exposed to higher pressures show similar volumes as control. B) Volume of nuclei measured after 38 hours under each given pressure, and then 18 hours of recovery. The pressure of 0.764 MPa corresponds to isotonic media (control). Nuclei that have been exposed to higher pressures and then allowed to recover exhibit the same volume as control, indicating that no permanent size alterations have occurred during osmotic compression.
(TIFF)

S2 Fig Volume measurement accuracy is not significantly influenced by increased z-step size. A) Volumes measured with z-step sizes of 1μm or 5μm as a function of volume measured with 0.5μm step size. A line of x = y is added as a visual guide. B) Ratio of measured nuclear volumes as a function of volume and z-step size, illustrating no significant loss in precision of volume measurements using a 5 μm step size. Here we plot the nuclear volume measured with a 1μm step (V1) divided by the volume measured with a 5μm z-step (V5), as a function of the “precise” volume measured with a 0.5μm step. The volumetric ratio is approximately 1 (ranging from 0.98 to 1.06, average = 0.02 +/- 0.06 error, single cells are presented here). This quantification demonstrates that the volumetric measurements in z using a 5 micron step are accurate.
(TIFF)

S3 Fig The pressure distribution appears spatially random in spherical MCAs. A) fluorescence image of nuclei in spherical MCAs, B) their measured volume, and C) their calculated pressure, of the nuclei relative to their position in the MCA in a 3D projection. As the MCA size increases in the fluorescence images shown in panel A, the nuclei volumes decrease in the plots in panel B, however no spatial pattern of volumes is visible, nor is there a clear pattern in the distribution of pressures in panel C. These data suggest that the volumes and pressures throughout spherical MCAs are similar, and that there is not a gradient from center to edge.
The perspectives in B) and C) are rotated to give a better visualisation of the 3D reconstruction of each MCA, and spatial dimensions are given in microns.
(TIFF)

S4 Fig The pressure distribution appears to increase at the periphery of elliptical MCAs. A) fluorescence image of nuclei in elliptical MCAs, B) their measured volume, and C) their calculated pressure, of the nuclei relative to their position in the MCA in a 3D projection. As the MCA size increases in the fluorescence images shown in panel A, the nuclei volumes decrease in the plots in panel B, and they appear more compressed near the edges of the elliptical MCAs. Their calculated pressures thus also suggested that nuclei at the periphery of the MCAs are under higher stresses, as shown in panel C. These data suggest that the volumes and pressures throughout spherical MCAs are different, with a gradient of increasing stress from center to edge.
(TIFF)
