# Supplementary figures and images for: Nuclei deformation reveals pressure distributions in 3D cell clusters
Source: PLoS One. 2019 Sep 12;14(9):e0221753. doi: 10.1371/journal.pone.0221753 (PMC6771309; doi:10.1371/journal.pone.0221753)

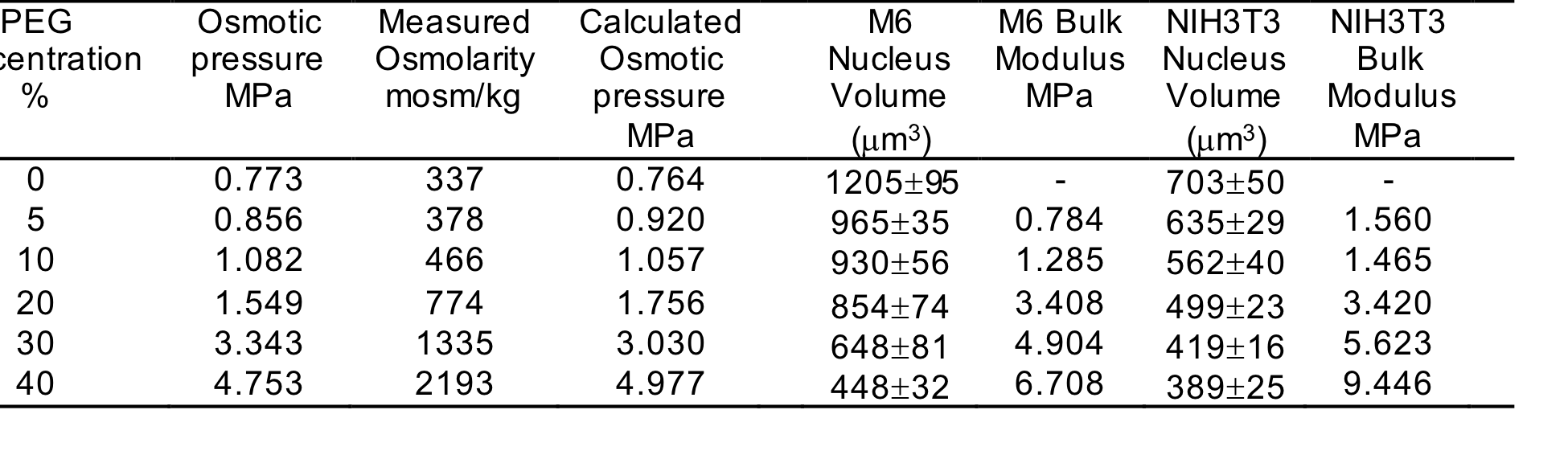

Supplement: S1 Table — Different PEG concentration w/v% in culture media (column 1), create varying osmotic pressure (column2). The osmotic pressure in column 2 corresponds to each PEG concentration based on the literature [1]. The osmolality of different PEG concentration (column 3) was measured by a freezing point depression osmometer, and their corresponding calculated osmotic pressures (column 4) is consistent with values previously reported in the literature. Columns 5&7 are the nuclear volumes for M6 and NIH3T3 cells under different PEG concentration, and columns 6 & 8 are their respective corresponding bulk moduli. (TIFF) [file pone.0221753.s001.tiff]

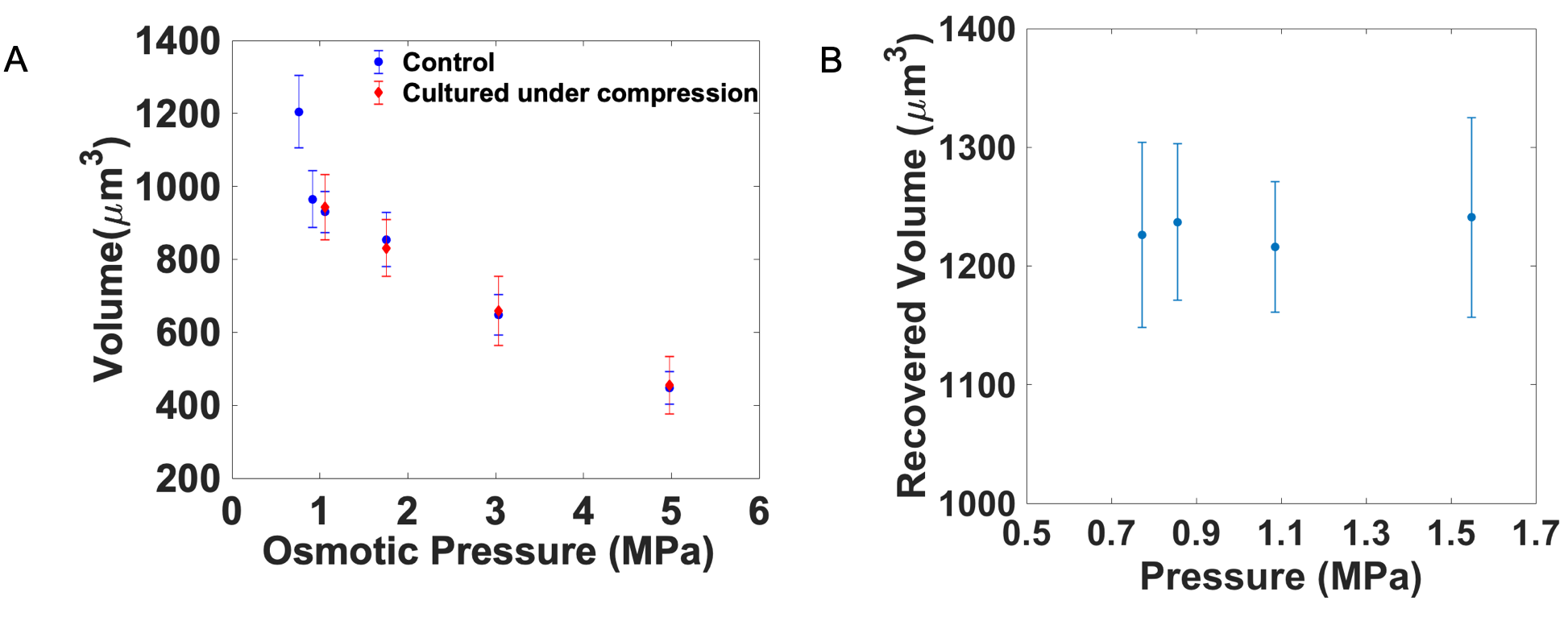

Supplement: S1 Fig — A) Volume of the nuclei measured after culturing cells under an osmotic pressure of 0.920 MPa for 21 days, without osmotic recovery, immediately exposed them to higher pressures. Nuclei that have been exposed to higher pressures show similar volumes as control. B) Volume of nuclei measured after 38 hours under each given pressure, and then 18 hours of recovery. The pressure of 0.764 MPa corresponds to isotonic media (control). Nuclei that have been exposed to higher pressures and then allowed to recover exhibit the same volume as control, indicating that no permanent size alterations have occurred during osmotic compression. (TIFF) [file pone.0221753.s002.tiff]

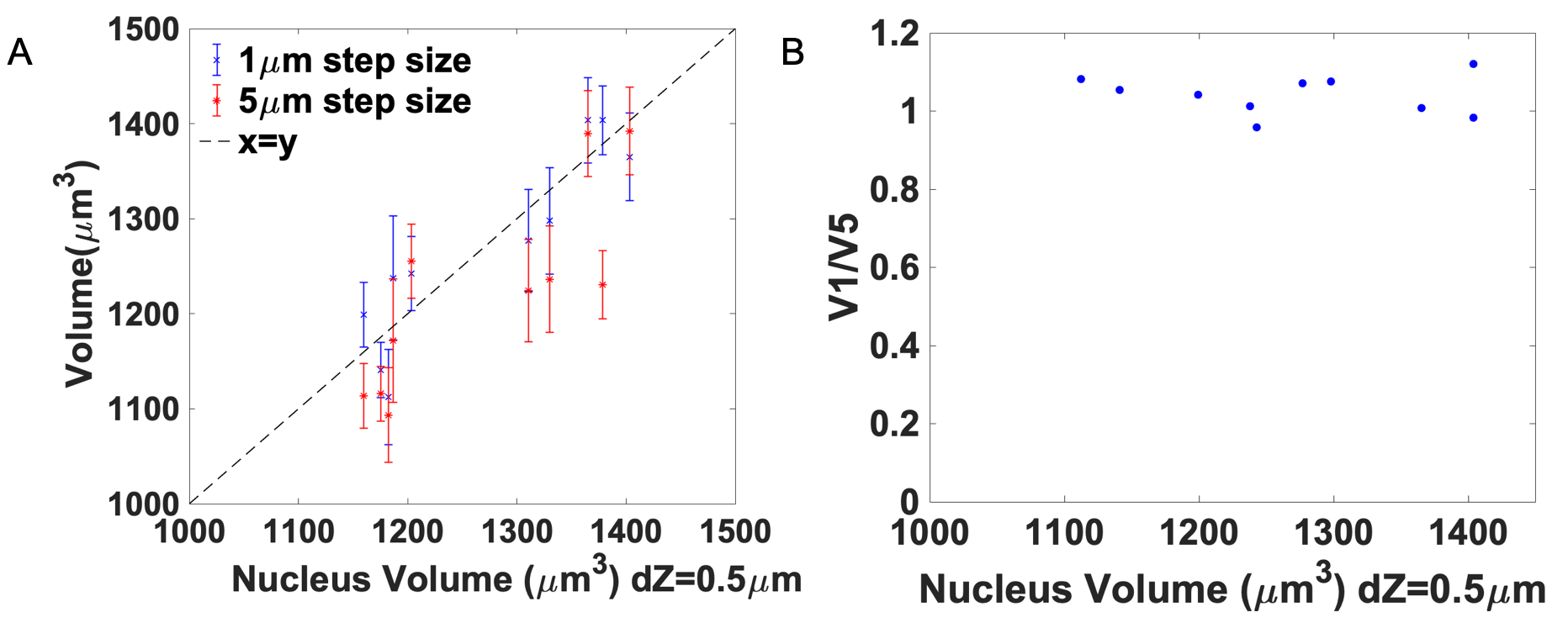

Supplement: S2 Fig — A) Volumes measured with z-step sizes of 1μm or 5μm as a function of volume measured with 0.5μm step size. A line of x = y is added as a visual guide. B) Ratio of measured nuclear volumes as a function of volume and z-step size, illustrating no significant loss in precision of volume measurements using a 5 μm step size. Here we plot the nuclear volume measured with a 1μm step (V1) divided by the volume measured with a 5μm z-step (V5), as a function of the “precise” volume measured with a 0.5μm step. The volumetric ratio is approximately 1 (ranging from 0.98 to 1.06, average = 0.02 +/- 0.06 error, single cells are presented here). This quantification demonstrates that the volumetric measurements in z using a 5 micron step are accurate. (TIFF) [file pone.0221753.s003.tiff]

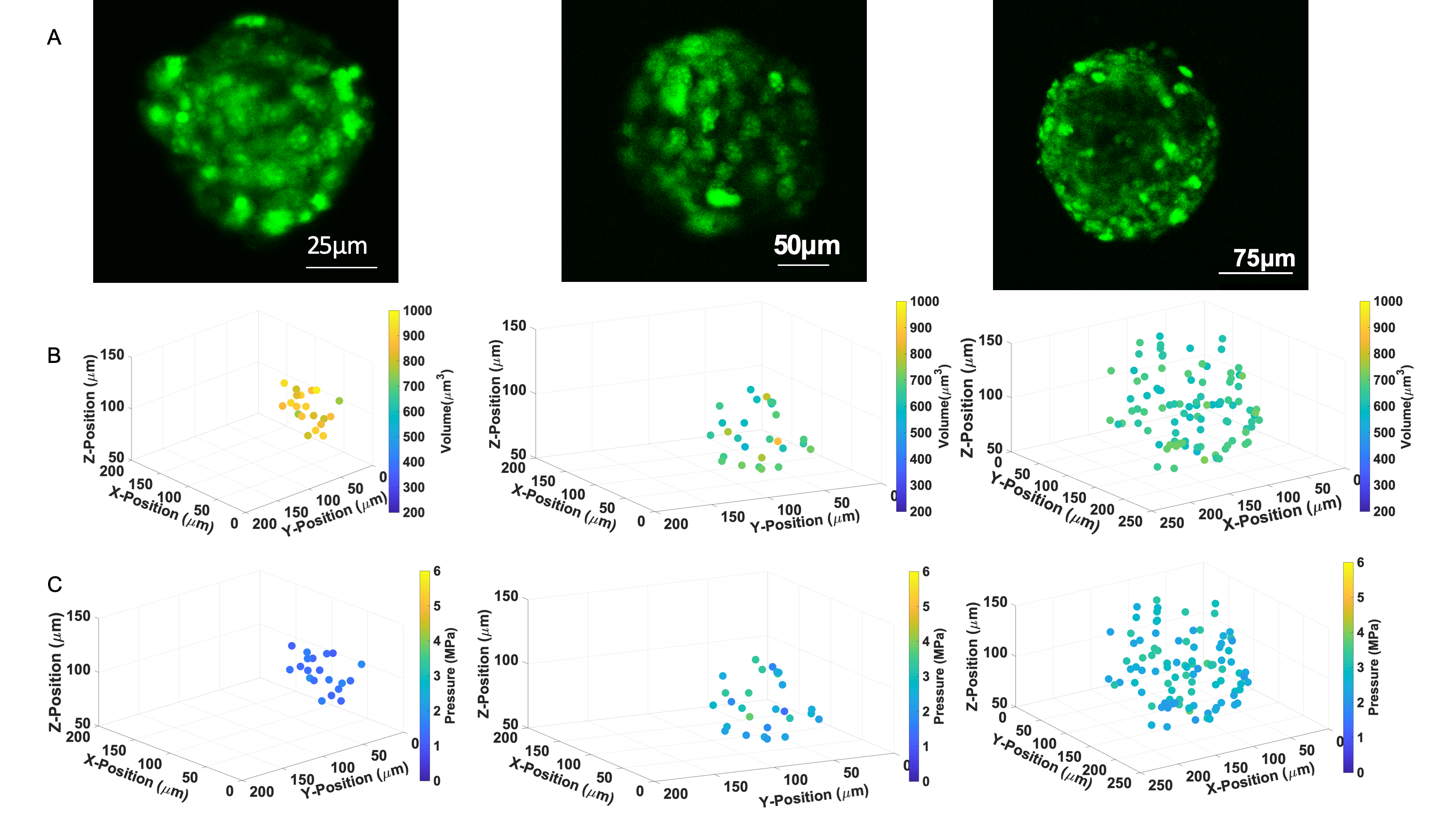

Supplement: S3 Fig — A) fluorescence image of nuclei in spherical MCAs, B) their measured volume, and C) their calculated pressure, of the nuclei relative to their position in the MCA in a 3D projection. As the MCA size increases in the fluorescence images shown in panel A, the nuclei volumes decrease in the plots in panel B, however no spatial pattern of volumes is visible, nor is there a clear pattern in the distribution of pressures in panel C. These data suggest that the volumes and pressures throughout spherical MCAs are similar, and that there is not a gradient from center to edge. The perspectives in B) and C) are rotated to give a better visualisation of the 3D reconstruction of each MCA, and spatial dimensions are given in microns. (TIFF) [file pone.0221753.s004.tiff]

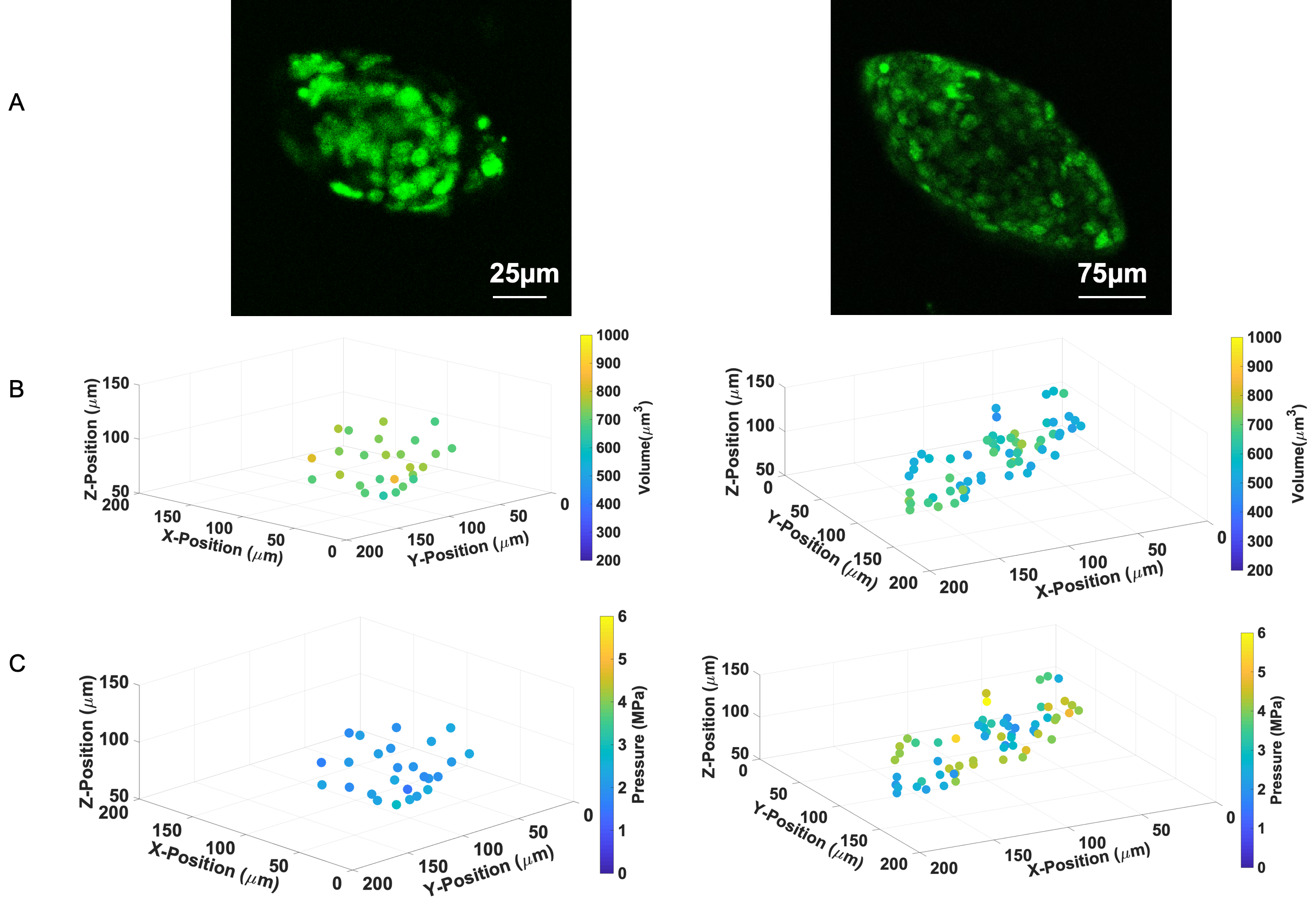

Supplement: S4 Fig — A) fluorescence image of nuclei in elliptical MCAs, B) their measured volume, and C) their calculated pressure, of the nuclei relative to their position in the MCA in a 3D projection. As the MCA size increases in the fluorescence images shown in panel A, the nuclei volumes decrease in the plots in panel B, and they appear more compressed near the edges of the elliptical MCAs. Their calculated pressures thus also suggested that nuclei at the periphery of the MCAs are under higher stresses, as shown in panel C. These data suggest that the volumes and pressures throughout spherical MCAs are different, with a gradient of increasing stress from center to edge. (TIFF) [file pone.0221753.s005.tiff]
